# From lab to mass production: a policy for enabling the licensing of mRNA vaccines

**DOI:** 10.3389/fpubh.2023.1151713

**Published:** 2023-05-18

**Authors:** Andreas Panagopoulos, Katerina Sideri

**Affiliations:** ^1^Department of Economics, Knowledge Transfer Office, TECHNIS, University of Crete, Rethymno, Greece; ^2^Department of Political Science and History, TECHNIS, Panteion University, Athens, Greece

**Keywords:** vaccines, mass production, WHO academy, know – how, barriers to entry, South African vaccine hub, technology transfer

## Abstract

Using the South African vaccine technology transfer hub supported by the WHO as an example, we show that the know-how needed to move mRNA vaccines from prototype to mass-production acts as an invisible barrier to market entry of mRNA vaccines. Overcoming this barrier relies on scarce human capital. In view of this scarcity and in preparation for the next pandemic, we propose broadening the scope of an existing WHO program, the WHO Academy, so that it coordinates knowledge diffusion initiatives by forming a systematized repository of know-how and a register of experts. As we explain, this proposal has an advantage in overcoming barriers to entry over current approaches of know-how acquisition.

## Introduction

Looking at the tail of a pandemic that introduced the word COVID into our global vocabulary and in preparation for the next one we must make an assessment of how humanity handled this emergency. On the one hand, the development of mRNA vaccines in such short notice is a notable achievement.^1^ On the other hand, our inability to manage their global diffusion and to produce enough and affordable vaccines for all is no doubt recognized as a major shortfall. In principle, bilateral licensing agreements could solve the problem. Yet, Moderna and Pfizer-BioNTech were frugal in offering product licenses to other producers (1, 2). Though the strategic considerations behind such thrift can only be guessed, following criticism by the World Health Organization (WHO) (3), pharmaceutical companies have proposed various initiatives. For example, Pfizer and Moderna have announced plans to build manufacturing facilities in Africa, and BioNTech has floated the idea of producing vaccines in *sea containers* shipped from Europe. But such proposals may run foul with developing countries’ regulations and may fall short of expectations as they rely on companies’ goodwill (4), and are faced with inadequate local production capacity, local regulatory inefficiencies and problems with viable long-term business plans (5).

The time tested way of necessitating licensing is by instigating imitation. This can be done via patent waivers,^2^ compulsory licensing^3^ or, if universities hold key patents, by low cost non-exclusive licensing (6). Alternatively, one can aid the redevelopment of a vaccine, jumpstarting competition, forcing the innovator to offer a license in order to avoid imitation (7). A WHO investment of more than 100$ million to a hub in South Africa attempted just that. It allowed a local firm (Afrigen) to re-create Moderna’s vaccine 6 months after Moderna’s vaccine had received FDA approval. Unfortunately, even though the WHO invited Pfizer-BioNTech and Moderna to participate in this hub and share their technology, they refused. Though often overlooked, there exists an invisible barrier to entry even when firms have re-created the technology. This barrier is none other than the know-how needed in moving from a prototype to mass-production. In Afrigen’s case, production has faced challenges and is currently envisioned to start after the pandemic has ended. Since Pfizer-BioNTech’s and Moderna’s market share is not threatened by Afrigen’s imitation, which involves producing a technology from 2020 a few years past its peak, their refusal to cooperate with the hub comes as no surprise.

In view of the above, the question we pose is *how can we overcome this barrier, allowing companies to reach a cooperative agreement with Pfizer-BioNTech and Moderna to share the technology, reducing the global vaccine deficit*? The WHO in cooperation with the National Institutes of Health (NIH) has already tried to endow Afrigen with the manufacturing expertise needed for swift mass-production. Yet, the task has proved challenging. We propose a simple solution: broadening the scope of an existing WHO educational program, the WHO Academy, to coordinate current knowledge diffusion initiatives in forming a systematized repository of know-how and a register of experts. Such a knowledge bank and community of experts that institutionalizes a way of overcoming the know-how barrier will precipitate the cooperative agreement Pfizer-BioNTech and Moderna refused to consider when asked by the WHO to assist Afrigen. This is a proactive solution that prepares us for the next pandemic.

## Barriers to entry

Though we often view ideas as non-excludable, in the sense that if they become known nothing stops their diffusion, certain embodiments of complex ideas are excludable. This is true even when their core knowledge is fully transcribed in the blue-prints we refer to as patents, whose role is to allow replication by people skilled in the prior art. Patents often omit peripheral yet relevant information. For example, despite patents describing how to derive the vaccine, they usually do not refer to the antigens produced (8). Furthermore, patents typically illustrate how to produce the vaccine in laboratory conditions. In reality the knowledge needed for mass-production is mostly uncodified and includes trade-secrets and tacit knowledge. We generally refer to this knowledge as know-how. One can view know-how as the unwritten tips regarding how to perform each step of a procedure (9). Since these tips are privy information, replicating the know-how for mass-production can pose a formidable challenge.

To visualize the web of techniques and trade-secrets needed to move from small batch production to mass-production let us illustrate the involvement of know-how in Moderna’s manufacturing process, focusing at the three most important steps. The first one is the *in vitro* stage of building the mRNA. At this stage, for stability, the mRNA must receive a cap on its one end and a tail at the other (10). Moderna’s platform applies internally invented screening approaches to identify proprietary sequences for the DNA region to be capped, in a way that allows the mRNA transcript to reliably initiate translation of the coding region. Without this knowledge sequence identification is impossible. Next, consider the purification that takes place after the *in vitro* stage has finished and the mRNA is generated. Since purification cannot rely on known methods of purification, which either cannot work on a large scale, or can only address specific impurities (11), internally developed techniques combining different purification methods are used. These methods are not public knowledge. Thirdly, during the last stage of production, which involves the encapsulation of the mRNA into lipid nanoparticles to form the vaccine particle (12), the four lipid components needed to encapsulate the mRNA are employed in undisclosed ratios (13). Unless these ratios are known production is handicapped.

Reliance on these unwritten tips is paramount even during the stage of putting together the factory floor. The bespoke specifications involved, which share few commonalities with usual drug manufacturing procedures, are challenging even for established producers. For example, Pfizer had to close down its Belgium manufacturing plant for 4 weeks until it was retrofitted to the purpose, with the overall validation of a single production site taking up to a year. This is due to the need for hyper-clean rooms and specialized capital equipment (bioreactors, filtration pumps etc.), as well as a capacity to employ extremely flammable inputs (14). Such machinery and its surrounding infrastructure are quite unlike Lego bricks that one can piece together. There is no manual one can rely on to attain the proficiency needed for this task. This is so even if the plant already has the specialized equipment, the needed space and the ability to produce. The reason is that even slight modifications to production may require product reviews because reorganizations of the factory space typically trigger new regulatory requirements (15). The challenges Pfizer faced when refurbishing its lipid production plant in Kalamazoo Michigan (enabling it to mass-produce lipids), a task it managed to finish in a record time of 9 weeks,^4^ is a case in mind.

The involvement of know-how is prominent even in quality-controls. The complexity of manufacturing and the biological nature of critical steps make production at times unpredictable. In fact, even small deviations in the manufacturing process can impact potency/purity. As a result assumptions regarding process integrity and product quality do not carry over from one batch to another (16). Thereby, there is a need to test results for each batch. These tests require an analysis of a variety of product specific biomarkers that require distinct techniques. Moreover, as quality-controls affect all stages of production and are not restricted to the final product, the harvested plasmids need to be compared with samples to make sure the coronavirus gene sequence has not changed. Linearized antigen genes need to be purified and tested again. After purification, the filtered mRNA must be tested repeatedly (17) to ensure its accuracy and the accuracy of gene sequence (18). Again, these procedures require a firsthand understanding of the DNA sequence, a comparison with standardized test samples, and sampling techniques that people outside the firm may not be in possession of.

### The acquisition of know how

In the absence of know-how there are two choices: acquisition or redevelopment. Redeveloping everything from scratch is hard, time consuming, and a duplication of innovative effort. Thus, focus should be on acquisition. Acquisition need not involve the original vaccine developer. Bits and pieces of the know-how are known to firms that face similar manufacturing issues, to professional institutions (e.g., the International Society of Pharmaceutical Engineering), or to organizations active in aiding the diffusing of vaccine technology, e.g., the Coalition for Epidemic Preparedness Innovations (CEPI). Such a spreading of expertise can allow governments to be proactive, planning ahead and preparing for a crisis.^5^

Acquisition is not straightforward, because what practically needs to be transferred is experience and experience is a commodity hard to transfer. This is because its transfusion involves the supply of experts in: manufacturing, engineering, logistics, quality-control and regulation, as well as skilled personnel such as, trainers, scientists, lab technicians, smart builders, maintenance crews, scientists, and above all capable leaders. For example, the transfer of know-how for the refurbishment of Pfizer’s Kalamazoo plant involved a team of 50 of its own experts across 20 different areas, including operations, product technology, quality assurance, procurement and planning. These experts are not only tasked with teaching and providing a framework and support (19). They must also handle misunderstandings, develop an appreciation of mutual responsibilities (1), and supervise the use of knowledge making certain it is fully shared.

If one needs to orchestrate multiple experts from different organizations the task becomes convoluted and time consuming. The transfer of know-how between Pfizer and Thermo-Fisher illustrates the complexity of the process. Despite Thermo-Fisher being involved in the fill-and-finish process of manufacturing (the least complicated part) the parties spent months exchanging information involving more than 500 top-secret files and at least 5,000 pages of documents. In all Pfizer employed a 24-person team in a process that lasted 7 months (2). This was because, quoting the WSJ, “*just transferring the knowledge of filling and capping the vials typically takes about 18 months and involves 10 stages, each consisting of hundreds of steps during which dozens of things can go wrong*.”^6^

Overall, despite being in possession of the recipe to create a vaccine, mass-production requires the diffusion of a wide set of skills. To appreciate the magnitude of the needed human capital, the head of Afrigen has stated their need of about 1,000 specialists.^7^ Even one of the world’s top vaccine manufacturers, the Swiss firm Lonza, required an additional 100 experts when it started producing vaccines for Moderna in 2021. Finding such experts is not easy. Consequently, the transfer of experience is faced with a bottleneck: the scarcity of human capital. Since in most cases such personnel is already employed, hiring and training the staff needed to transfer knowledge and maintain production is a challenge even for highly experienced manufacturers (8).

### Overcoming the barrier of know-how: the hub

The WHO addressed the problem of vaccine shortages by investing in institutionalizing vaccine development and production for local and regional use through a hub that acts as a center of excellence and training.^8^ Manufacturers from around the globe can use the hub’s expertise. Training will also be provided by the recently announced WHO Academy, a WHO Division. The Academy will provide mid-career training programs on health emergency preparedness, response and disease outbreak control. The center of the hub is a South African firm, *Afrigen* Biologics and *Vaccines and its local partners*. *The hub is connected to* a global network of companies called spokes, which will act as manufacturing centers. In strengthening and developing their expertise, the hub and the spokes will share technology, pre-clinical and clinical data, both between them as well as with third parties.

The primary issue the hub faced was the development of a vaccine. This challenge proved easier than anticipated. Afrigen managed to re-create Moderna’s vaccine by June 2021, mainly due to expert advice from the WHO, the Medicines Patent Pool (MPP) and scientists from around the world, including ones from the NIH that had conducted foundational work on mRNA vaccines. Afrigen did not re-engineer the vaccine. Following the advice of the WHO and the MPP it sought to re-create Moderna’s vaccine without infringing on patent rights. Plus, it abstained from seeking intellectual property (IP) protection. The focus on Moderna’s vaccine was due to Moderna’s pledge not to assert its patents during the pandemic. Plus, since Moderna’s R&D was funded by the NIH, it left a paper trail of knowledge such as the sequence of the vaccine, which was published by Stanford University in an open-source manner.

Nonetheless, scaling up production requires a lot more manufacturing innovation and this is where know-how becomes crucial. The fact that the manufacturing unit has become an important locus of innovation was recognized by the WHO when they invited Pfizer and Moderna to participate in this hub and share their know-how. When they refused to work with the hub, the WHO explicitly stated that this presents a major setback.^9^ In addressing this shortfall (in July 2022) the WHO turned to the US National Institute of Allergy and Infectious Diseases (NIAID), which agreed to share its expertise. The agreement focuses on diffusing knowledge relating to clinical trials, the needed Good Manufacturing Practices for the mass-production of the vaccine and its components, e.g., the lipid nanoparticle formulation. Despite such help, the prospects of mass-production still seem distant with early 2024 being the current estimate.

Two challenges remain. First, despite the fact that Afrigen sought to avoid infringing patent rights, this is not to imply that Afrigen is not liable to face infringement accusations. After all, Moderna has filed for COVID-19 related patents in South Africa and has stressed that its pledge applied for the duration of the epidemic only. As Afrigen’s CEO has admitted “*We have full freedom to operate, an exemption under the Bolar Exemption* [i.e. research exemption] *in IP law. So up to phase 3 clinical trials we are completely legal, and we do not need any permission. Once that product is commercialized and there are IP constraints, we need to get a voluntary license for that*.”^10^ How is voluntary licensing to materialize is not obvious. The mRNA vaccine and its components are protected by a patent web that belongs to the University of Pennsylvania and its main licensee Cellscript, as well as firms like Moderna and Pfizer. Moreover, vital technologies such as lipid technologies belong to Acuitas, Arbutus and Genevant. Some of these firms are aggressively litigious (e.g., Arbutus) and some are already locked in patent infringement suits. Plus, Moderna and Pfizer view Afrigen as a competitor who can produce other mRNA products in the future. Any infringement suit, either in South Africa or in any of the countries where the spokes are located can further delay production.

Regulatory approval equally seems distant. Animal tests started in October 2022, and human clinical trials are expected to begin in 2023, with regulatory approval from the Food and Drug Administration (FDA) or the European Medical Agency (EMA) expected in 2024. As Martin Friede (coordinator of the WHO Initiative for Vaccine Research) admitted, this waiting period can be abridged only “*if companies with approved COVID vaccines or late stage clinical data shared their technology and data with the consortium*.”^11^ Yet, the hub is not in an immediate need for an FDA/EMA approval. It has the option to opt for an Emergency Use Listing (EUL). The EUL is a WHO procedure for reviewing the quality, safety and efficacy of unlicensed vaccines during public health emergencies. It is a risk–benefit assessment to decide if vaccines can be used outside clinical trials. The EUL opens the door to countries that lack robust regulatory systems and need to rely on WHO’s review process in expediting their own regulatory approval. The prerequisite for an EUL is for a country’s regulatory system to have reached maturity level three (indicating that it ensures the quality, safety, and effectiveness of vaccines manufactured and distributed in the country), which South Africa reached in October 2022. Consequently, upon securing an EUL, Afrigen can market its vaccine in South Africa and possibly other African countries.

Against this factual background how would one rate the hub? Though, it is evident that the hub can bypass know-how problems, mass-production is expected when COVID will no longer be of concern. Yet, one could still argue that the hub puts together the infrastructure needed for future epidemics. Viruses as well as technologies are not static, they evolve. Unless a new epidemic shares many commonalities with this one, the technology needed to manufacture a vaccine will be different and so will the underlying know-how. Another thing that is also non static is funding opportunities. Once an epidemic terminates, assuming the WHO views the hub as vaccine development project only, funding must be channeled to other causes. Unless the WHO can continue investing its limited funds on the hub and its spokes, any shift in priorities will hinder capacity building.^12^ On account of the above, the hub seems insufficient for the task.

### The way forward

Epidemics are dynamic phenomena. As the “measurable” cost of delaying vaccination is enormous, expeditious reaction is the key-word in addressing epidemics. If the hub can only produce belated solutions, how can we improve this model and ensure a timely reaction for epidemics to come? Looking at mass-production, as long as the culprit behind mass-production delays is lack of know-how, addressing the issue must no doubt involve securing the needed expertise. There exist institutions with such know-how. For example, NIAID, or the non-profit International AIDS and Vaccine Initiative (IAVI) research center, can offer much of the science needed in re-creating a vaccine. Moreover, international initiatives such as WHO’s Local Production and Assistance Unit, the Manufacturing Task Force the COVID-19 Vaccine Global Access (COVAX), or the UN’s International Vaccine Institute can offer help with potential business models, establishing the required infrastructure, workforce training, Good Manufacturing Practices, regulatory needs, network formation etc. The same is true with manufacturer alliances or educational initiatives such the Pan African University, or the Developing Countries Vaccine Manufacturers Network. However, despite the fact that they can collectively offer much of the needed know-how, these initiatives are run by an array of organizations [such as the WHO, UN, EU, UNISEF, the NIH, COVAX, CEPI, the Global Alliance for Vaccines and Immunization (GAVI), the Bill & Melinda Gates Foundation etc.] which do not coordinate their actions.

In overcoming the problem we do not need to reinvent the wheel. The hub already bundles some interesting ideas. The WHO Academy stands out. The Academy is a mid-career training program aimed at health workers, managers, public health officials and policy makers. Its flagship programs address vaccine equity, universal health coverage and health emergencies. Yet, nothing stops the Academy from expanding its bearing, becoming an umbrella organization that coordinates with the aforementioned initiatives in forming a repository of related expertise and a register of all suitable experts, while acting as a facilitator of the provisions needed to accommodate their services. Such a *college of dexterity* will be in a position to overcome all barriers related to the transfer of know-how both at the vaccine development stage and at the manufacturing stage.

The list of up-to-date expertise that such a *college* should be able to endow the hub with must depend on the type of the virus causing the pandemic. Nevertheless, even the main generic skills needed are considerable. For example, in terms of engineers the hub will need people who have the latest skills in: Vaccine Formulation, Compliance and Verification, Digital Plant Automation, Information Technology Infrastructure, Cloud Operations, Reliability and Maintenance, and Packaging. Looking at experts/analysts, the hub will find it hard to function without experts in: Pharmacovigilance, Cybersecurity, Asceptic Processing, Active Pharmaceutical Ingredients, Injectable Packaging, Visual Inspection Technology, Parenteral Operations, Rapid Turn Laboratory Analysis, Biostatistics and Programming, Global Animal Welfare, Environment Health, Safety and Security, Quality Assurance Distribution, Bioprocess and Formulation. The overall list must also include managerial personnel to coordinate various functions related to pharmaceutical production such as: Drug Product Training, Technical and Regulatory Affairs, Customer Support, Quality Operations, Process Safety, Risk Management, Regulatory Affairs, Supply Chain Operation, Nonclinical and Early Development.

At first look, institutionalizing a community of experts under the auspices of the WHO does nothing more than to hasten mass-production. It fails to curb the hub’s IP problems or help it obtain a more general regulatory approval than an ELU. However, there is a considerable difference between a firm that can manufacture a vaccine is 2024 and one that does so in 2021. In the first case, a latecomer has managed to manufacture a vaccine that (by this time) has progressed passed its 2020 form and is in need for updated know-how (and regulatory approval) in order to be produced in its latest incarnation. Plus, this firm lacks a patent portfolio that can be used as a bargaining chip (20) in obtaining a cross-licensing agreement to allow manufacturing absent infringement. In the second case, overlooking the fact that during times of crises regulators are more flexible in their understanding of what constitutes infringement, the firm swiftly over passed technical challenges and is well positioned to address any future requirements, e.g., viral mutations. The second firm must be viewed by Moderna and Pfizer as a competitor, while the first one is just a copy-cat. Such agility in over-passing technical challenges may free many birds with one key, allowing Afrigen to overcome its regulatory problems and looming IP issues.

Corporations dislike a vacuum competitors can fill at their expense. In view of prospective imitation they have two choices: either compete and share the market, or license the product, share the market, and profit from royalties. In present terms this dilemma runs as follows. Should Pfizer-BioNTech and Moderna compete with a firm that has the potential to market a variant of their vaccine to low-and-middle-income countries,-a market they largely neglected, or should they instead license and benefit from royalties? Economic theory, and common sense, predicts the latter (7).

There is also a non-pecuniary reason to agreeably resolve competitive actions between the hub and the firms through a licensing agreement: *stability*. Amicable solutions lead to stability (21), which increases profitability. This is especially so if atypical outsiders, who command sizable “soft power” (e.g., charities, NGOs, international bodies etc.), are party to this conflict. Despite their indirect involvement in the product and factor market, such outsiders have the potential to inflict firms with disproportionate and unanticipated damage (22), destabilizing their operations. Thereby, considering the involvement of the WHO and the sizable power it yields, it may be best to cordially find a solution that serves the interests of all.

Accounting for the above, a WHO Academy that institutionalizes a way of overcoming the know-how barrier will precipitate the cooperative agreement Pfizer-BioNTech and Moderna refused to consider when the WHO asked them to assist Afrigen. Such a solution no doubt allows Afrigen to manufacture an already approved vaccine in a way that avoids IP conflicts. Furthermore, low-and-middle-income countries will be able to procure vaccines at a much smaller price than the one charged by Pfizer, which ranges from $6.75 (the price charged to the African Union) to $28 (the price Israel paid) and is expected to increase after the pandemic.

**Figure 1:**
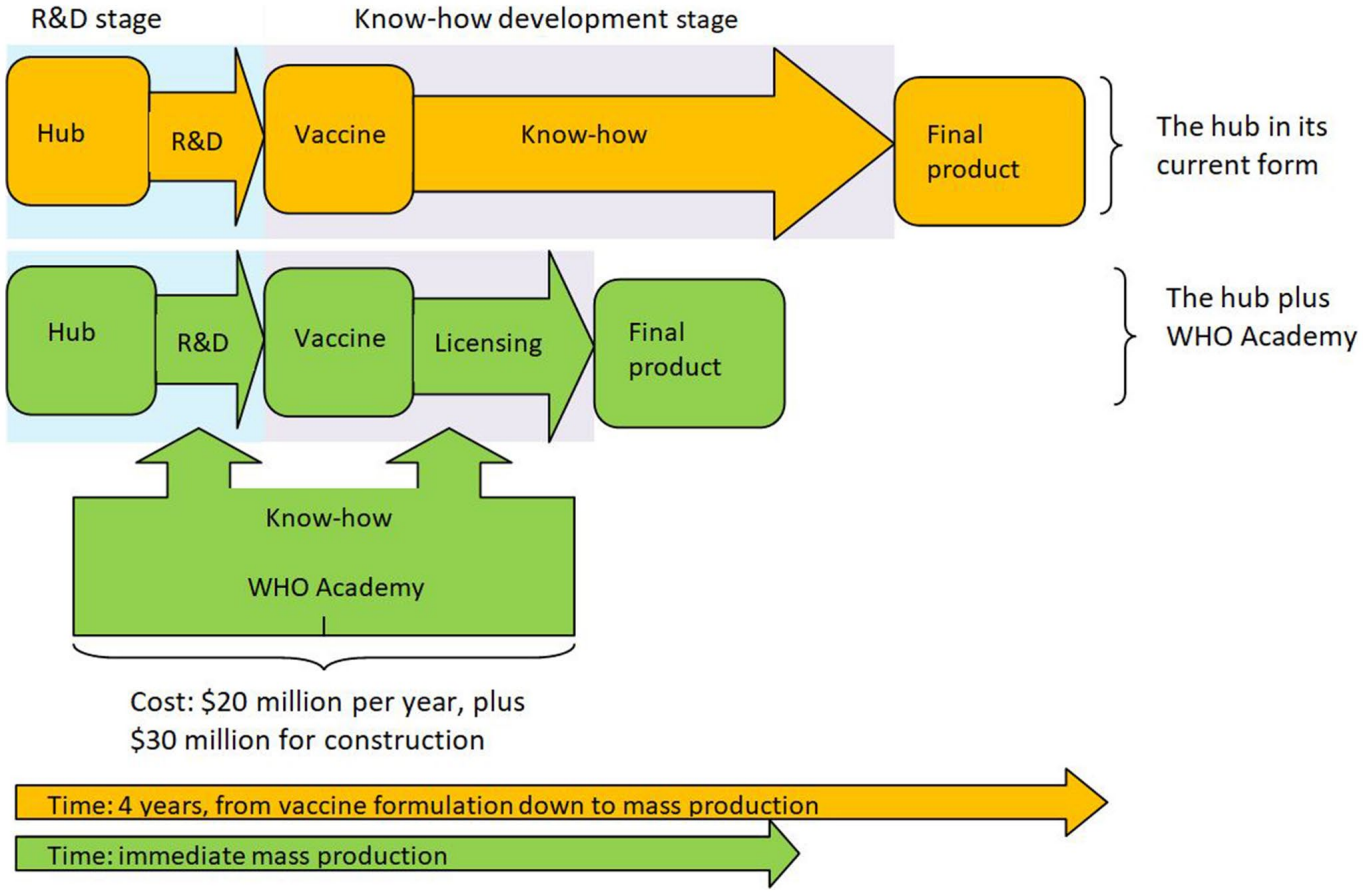
The hub plus the WHO Academy.

What will this price be? The current marginal cost of the vaccine is estimated at $1.20 (10). Though one should expect Afrigen to have a smaller production cost, one cannot know in advance the per unit royalties Afrigen will have to pay for licensing the technology. Yet, due to the involvement of the WHO, it is safe to assume that the licensing fee will be fair, reasonable and non-discriminatory (FRAND). This usually means that the licensors will be compensated at a per unit price that is at least equal to their marginal cost, i.e., $1.20. Thus, due to Afrigen’s small marginal cost, one should expect the overall cost per dose (i.e., Afrigen’s marginal cost plus royalties of $1.20) to be less than double this amount (i.e., $2.40), which makes it far cheaper than the lowest price Pfizer has offered. This means that the vaccine can be affordably priced for all African countries, making the hub and the accompanying WHO Academy a viable solution to the problem. Noting the measurable economic benefits from vaccination (23), the increased benefits that the hub can achieve outweigh all costs. In view of this we depict the hub plus the WHO Academy in Figure 1.

### Comparisons

How does this policy compare with other methods that aim to transmit knowledge? There are three types of policies that can be used for this purpose: waivers/compulsory-licensing, preconditions set by the original innovator, and partnerships. Partnerships, which can allow for either full or partial knowledge transfer, constitute the only method put to the test.

Regarding full transfer, even though various schemes involving comprehensive knowledge transfer via the creation of manufacturing sites in Africa have been proposed (by Pfizer, Moderna, and BioNTech), none has fully materialized. Even Moderna’s well-advertised $500 million investment in a manufacturing site in Kenya had to be considerably scaled down, and the beginning of construction postponed until March 2023. Though it is impossible to assess the reasons for this, the already outlined complexities in creating a production plant, the substantial regulatory requirements for building such sites (24), and the fact that demand for vaccines plummets when the pandemic ends, no doubt make such investments risky.

If full knowledge transfer partnerships are not available, the only partnerships the hub can pursue must rely on agreements that consent to partial knowledge transfer. This advances a fragmented approach to the problem, which can work only if all the pieces of the puzzle can be successfully brought together in creating a coherent whole. For example, even though Afrigen has a partnership with the NIH and NIAID that will help with clinical trials and material transfers, this agreement will not provide Afrigen with the skills needed for the development of nanoparticle formulations, or the knowledge involved in super-cold production chains. In acquiring such knowledge Afrigen had to independently partner with Curepath and with Univercells, respectively. However, this expertise is still not enough to allow for mass production and additional knowledge is needed, e.g., in filtering, in managing flammables, in fill and finish procedures, in distribution etc.

Though there is little doubt that in time Afrigen can gradually acquire all the needed expertise, partial knowledge transfer agreements can provide a solution only if there already exists a good understanding of which skills/technologies will be needed. Accordingly, if we are to avoid being witnesses to the same play, we need to ascertain that there exists a mechanism whose job is to know in advance what technologies may be required for the purpose. This apparatus will allow the organization to prioritize the acquisition of relevant knowledge and skills, cementing all the technologies together in a timely fashion. In the absence of such a mechanism any piecemeal approach to the acquisition of knowledge can only be coordinated via *learning on the job*. It is this inefficiency the WHO Academy aims to remedy.

Turning our attention to waivers/compulsory-licensing in June 2022, the WTO Ministerial Conference agreed on a limited-time waiver for COVID-19 vaccines for specific countries. Under a waiver or a compulsory-license, governments may authorize the non-exclusive use of patents by a domestic manufacturer without the holder’s consent. Apart from being a reactive approach to the problem, there is an evident issue with such measures; one cannot be forced to share sizable technical expertise that is unknown to outsiders. Had the requisite know-how been simple this would not have been a problem. One could envision replicating the relevant Japanese experience regarding the institutionalized rotation of engineers between firms, which allows firms to be up to date by having access to what others have accomplished. Yet, in this solution there is (a) reciprocity, and (b) limited staff rotation. In the absence of reciprocity, or any other substantial motive, it is difficult to see how to persuade firms to voluntarily contribute to such extensive exchange of knowledge, which involves many hard to find experts. Additionally, waivers are faced with a moral hazard issue. Even if the firm is “persuaded”, due to the complexity of the process it is impossible to figure out who is to blame in case of failure. Due to the inability of waivers or compulsory-licenses to offer an alternative means of production it comes as no surprise that firms like Pfizer behaved like monopolists, either insisting on exuberant prices for their vaccine, or offering half-backed bilateral manufacturing solutions, like the one involving production in sea containers (4).

Preconditions in licensing innovations invented at universities, endows universities with a stick with which they can proactively enforce ethical considerations. For example, universities can ask for affordable prices (25) or an adequate supply of the final product (6) as a condition for licensing their patents. Failure to do so could lead to non-exclusive sharing of the technology. Since for vaccination these preconditions can only be met via an expansion of production, the licensor can indirectly force firms to share their technology. It is straight forward to extend this idea beyond universities as governments can equally set similar terms in exchange for R&D funding (26). One can envision similar conditions being set by *push* policies that aim to promote R&D, such as prizes, or *pull* policies that seek to facilitate vaccine procurement, e.g., advance market commitments. Yet, there is a vital issue that needs to be addressed. It is not a credible threat. During a pandemic you cannot threaten a vaccine producer to withhold a key license. Such a threat, if enforced, would bring production to a standstill and would disrupt the value chain, breeding uncertainty about what belongs to whom. Even if the key technology is licensed to others, it will take time until they self-develop the needed know-how. To rephrase, *in a joust you do not take your champion out of the arena in order to train a novice*. We summarize the main elements of the above comparison in Table 1.

**Table 1:** Comparing types of technology transfer.

Type of tech-transfer	Temporality	Exclusivity	Disadvantages
Preconditions	Proactive	Non exclusive	Not a credible threat
Waivers and compulsory licensing	Reactive	Non exclusive	Difficulty in enforcing the exchange of know-how
Full or partial partnerships	Reactive	Exclusive	Relies on firm strategy. No means for governments to impose their will.
Hubs	Proactive	Non exclusive	Difficulties in mass production
Hubs plus academy	Proactive	Non exclusive	Reliance on a coordinator

### Is this solution feasible and sustainable through time?

We have up to now assessed how a hub plus Academy would have managed to mass produce a vaccine at the height of the pandemic. In economic terms we have displayed the static efficiency of this policy by explaining how it can presently address market failure (27) by supplying vaccines to markets largely unattended by other vaccine producers. The question we now seek to address is one of dynamic efficiency. In emergencies to come, will this policy be able to deliver on its promise? And if the hub is expected to mass produce drugs during emergencies how should it sustain its existence in the meantime?

Though at the moment the emphasis is on vaccines, upon the termination of the pandemic this market will surely stagnate. This need not imply that the interest on mRNA technologies will stagnate as well. mRNA is a platform technology that can find multitudes of uses. If we are to assess the dynamic efficiency of the hub the first question we need to ask ourselves is: who else stands to benefit (or loss) from the knowledge the hub possesses and how are they expected to interact with the hub in the near future? If the answer to this question involves powerful incumbents and sizable markets any ensuing rivalry will require the hub to tread a fine line.

The most promising forthcoming uses of mRNA technology seem to be directed toward oncology. This market is currently valued at $148 billion, and it is expected to reach $288 billion by 2030. The stakes in this market are much greater than in the market for vaccines. The main players (Roche, Novartis, Pfizer, Johnson & Johnson, Bristol Myers Squibb) have invested $63 billion in their overall R&D during 2022 alone. Moreover, they hold at least 2000 patents on technologies related to oncology.^13^ As these agents have ongoing relationships with the practitioners and the academics needed in supplying knowledge to the WHO Academy, they can influence their decisions (28, 29) to support or not the hub. This is equally so for the national agencies (30) and charities (31) that can assist the hub. Furthermore, as these companies control many of the inputs needed in employing mRNA technologies, they have the capacity to create supply-chain restrictions (1).

These incumbents must no doubt view the hub with suspicion. After all, its newly acquired expertise can be redirected to the development of new drugs, or new techniques that further advance the potential of mRNA technology, in which case competition between the hub and incumbents should be nigh. The hub would be ill advised if, in its strategic planning, it does not account for the power such groups yield. This is because theory prescribes that due to their joint interests in this market (despite being competitors), they can unite their actions against entrants (32, 33), using their might to deprive the hub from valuable human capital (34) and its adjacent knowledge flows. In such an occasion, despite WHO’s best intentions, the joint actions of such common-interest groups (35) have the potential to decrease social welfare (36) by limiting the capacity of the hub to achieve its purpose. Accounting for the sizable power these groups yield, the precondition for the continuous success of the hub must surely be absence of rivalry between the hub and these firms.^14^

This is not to say that the hub should not try to fulfill its purpose and prepare for the next emergency. It simply means that it should not (in the interim period) compete in licensing new products/techniques. After all, experience from the current pandemic suggests that when the time is ripe for the hub to compete (during a crisis), the power that incumbents yield will not affect the hub’s operations. To explain this point, for example, judging from the help the hub has received from national agencies, international bodies, charities and experts, when the need arises (i.e., a crisis) they will help the hub acquire all needed knowledge, irrespective of any ongoing relationships with incumbents. Furthermore, as Operation Warp Speed has indicated, during crises governments intervene and act as coordinators, directing industrial *production so that there is no shortage of inputs related to manufacturing and R&D. Such initiatives, which were not limited to the US*,^15^ *illustrate that during emergencies incumbents cannot easily restrict the supply of* the inputs the hub may need. By the same token, Moderna’s pledge not to enforce IP rights shows that during pandemics it is easier for the hub to tinker with patented knowledge.

To summarize, considering that incumbents cannot impose their will on the hub during emergencies, as long as the hub continues enhancing its capacity to recreate and mass produce future medications without actively competing in the market, yet fully springing into action when the time arises, rivalry will be minimal. In the meantime, nothing stops the hub from negotiating contracts for future vaccines with charities, such as GAVI, or with governments, strengthening its hand in bargaining a good contract to manufacture vaccines for low-and-middle-income countries when a future emergency is upon us.

One possible issue with this strategy is the structure of the hub. The hub operates as a loose confederation of allied interests, with Afrigen at its core and many spokes in various countries acting as local producers. As these spokes have their own interests, the availability of a platform technology can be seen by some of these spokes as the gateway to the production of valuable pharmaceutical products, leading to a conflict. Nevertheless, a first look at the spokes (e.g., Argentina’s Sinergium Biotech and Brazil’s Bio-Manguinhos/Fiocruz) gives the impression of small companies that have limited R&D experience, whose main function is to produce vaccines under license. It is hard to envision such firms taking the step to compete with behemoths while lacking full control of their IP.

A more imminent problem is the interim financing needed in order to keep the hub and the WHO Academy alive until their skills are needed. Starting with the hub, Afrigen and the spokes will not require funding. Prior to their involvement with the hub they were functioning firms that specialized in vaccine production. Their newly acquired know-how should no doubt enhance their capacity to expand the scope of their operations, allowing them to remain profitable. This is not so for the Academy. As the Academy will need to be financed by the WHO the question of merit is: *how much will this cost*?

This question is difficult to answer. Nevertheless, as the Academy is broadly similar to a specialized medical college, one can try and see how much such institutions cost to create and run. A recent estimate by the Wall Street Journal^16^ places the annual cost for staff and faculty in the $15–$20 million range and the cost of the buildings in the range between $50 and $100 million. Outside the US prices can be much smaller. In South Africa in particular, the cost of building a full university (the University of Mpumalanga, established in 2014) is expected to cost about $56 million in capital and operational costs during the first 10 years. A smaller university, Sol Plaatje University, is expected to cost $37 million.^17^ From own experience a medical school in Greece (without the university hospital) that is staffed with about 200 personnel, costs about $20 million to run per year, while the cost of construction ranges from $15 to $30 million. Bearing in mind that the WHO’s budget for 2022 was $6.72 billion, and considering the overall socioeconomic cost of the pandemic (37), especially in continents like Africa (38), and the economic benefits from vaccination (23), the modest cost of running the Academy as a precaution for emergencies to come seems like a bargain.

Despite the above prescribed caution to instigate rivalry, no discussion on the hub’s potential would be full unless it accounts for its capacity to do research and produce remedies for neglected diseases (39). After all, by definition, such diseases are outside the scope of operation of incumbents. Therefore, at first look, the emergence of a conflict seems unlikely if the hub decides to use its technical expertise for such a purpose. Yet, this is a delicate matter because, even though the final product may be out of the incumbents’ sphere of interests, this may not be so for the techniques developed for this purpose. Accordingly, as this topic is outside the paper’s area of interest, and noting the need for a comprehensive analysis, we plan to address it in forthcoming research.

## Conclusion

We have identified a barrier that limits the production of vaccines even when the technology is available. This barrier is the know-how needed for mass-production. Furthermore, we have explained how an Academy that acts as a repository of expertise and a registry of specialists, can enhance the capacity of hubs to address this barrier in a non-exclusive way that sponsors global vaccine production. This is a proactive approach that, unlike preconditions and hubs, is both credible and solves the problem of mass-production. Plus, as long there is an international body that coordinates the hub and the Academy, it can attain the needed know-how in a way that reactive methods like waivers fail to do. Equally, this proposal is better than bilateral deals, because it does not rely on firm strategy. Thereby, there is no need to persuade firms to align their strategic objectives with the greater good.

## Author contributions

All authors listed have made a substantial, direct, and intellectual contribution to the work and approved it for publication.

## Conflict of interest

The authors declare that the research was conducted in the absence of any commercial or financial relationships that could be construed as a potential conflict of interest.

## Publisher’s note

All claims expressed in this article are solely those of the authors and do not necessarily represent those of their affiliated organizations, or those of the publisher, the editors and the reviewers. Any product that may be evaluated in this article, or claim that may be made by its manufacturer, is not guaranteed or endorsed by the publisher.

## Footnotes

^1^mRNA vaccines prove more efficacious, safe and scalable in comparison to conventional techniques.

^2^A waiver temporarily relinquishes the intellectual protections provided by the WTO or governments.

^3^Compulsory licensing allows a government to permit the production of a patented product/process without the consent of its owner.

^4^ https://www.pfizer.com/news/articles/shot_of_a_lifetime_how_pfizer_developed_its_own_raw_materials_to_ensure_a_steady_supply_for_the_covid_19_vaccine

^5^It is worth noting that there is no legal requirement in International IP law compelling the transfer of know-how.

^6^Jared S. Hopkins, “Key Staffers Aid Global Vaccine Rollout,” Wall Street Journal, August 20, 2021, https://www.wsj.com/articles/pfizers-global-covid-19-vaccine-rollout-depends-on-two-expert-staffers-11629464010?page=1

^7^ https://healthpolicy-watch.news/has-south-africa-made-modernas-vaccine-scientists-are-not-yet-sure-because-there-has-been-no-tech-transfer/

^8^Another way the WHO chose to address the problem of shortage was the creation of COVID-19 Vaccines Global Access (COVAX). Yet, COVAX has failed for a variety of reasons see https://www.washingtonpost.com/world/2022/03/22/covax-problems-coronavirus-vaccines-next-pandemic/

^9^ https://healthpolicy-watch.news/has-south-africa-made-modernas-vaccine-scientists-are-not-yet-sure-because-there-has-been-no-tech-transfer/

^10^ https://www.voanews.com/a/africa-s-first-mrna-covid-19-vaccine-produced-/6426738.html

^11^ https://www.reuters.com/world/africa/approval-covid-vaccine-made-south-africa-could-take-3-years-who-says-2022-02-04/

^12^Funding can continue if the WHO views the hub as a developer of future mRNA technologies. Yet, the WHO has yet to provide such indications.

^13^A patent search contacted in March 2023, using only the term oncology, found 2063 patents that have yet to expire by these firms.

^14^This suggestion is on par with the expert opinion Médecins Sans Frontières (MSF) has offered the hub, advising it to avoid using patented technologies for purposes other than research, and to re-engineer the technologies it needs in a non-infringing fashion; see: https://msfaccess.org/removing-intellectual-property-barriers-covid-19-vaccines-and-treatments-people-south-africa

^15^EU’s COVID-19 Clearing House for medical equipment played a similar role in facilitating manufacturing capacity.

^16^ https://www.wsj.com/articles/BL-HEB-2340

^17^ https://www.businesslive.co.za/bd/national/education/2013-07-26-government-projects-cost-of-two-new-universities/
